# Use of network meta-analysis in systematic reviews: a survey of authors

**DOI:** 10.1186/s13643-015-0174-4

**Published:** 2016-01-19

**Authors:** Andrew W. Lee

**Affiliations:** Department of Continuing Education, University of Oxford, Rewley House, 1 Wellington Square, Oxford, OX1 2JA UK

**Keywords:** Research design/trends, Mixed treatment comparison, Evidence-based medicine, Indirect comparison, Meta-analysis

## Abstract

**Background:**

The reporting of network meta-analysis in systematic reviews has increased rapidly since 2009. This qualitative study was undertaken to identify authors’ perceptions of the use of these methods and of what standards for conduct and reporting should apply.

**Methods:**

This is a survey of authors of systematic reviews reporting network meta-analysis.

**Results:**

The response rate was 32 % of the authors contacted, with these authors responsible for 34 % of the fully published systematic reviews identified within the period searched. Almost all authors would use the method again. Elements of reporting standards were proposed. Responses revealed some tensions between the view that use of network meta-analysis should be more easily accessible, particularly in the form of software tools, and concern that there is some inappropriate use of the methods, which wider use and greater accessibility could exacerbate.

**Conclusions:**

Authors demonstrated strong support for adoption of standards for conduct and reporting. The elements of reporting standards proposed are consistent with those included in the 2015 Preferred Reporting Items for Systematic Reviews and Meta-Analyses (PRISMA) extension statement. Adoption of standards for conduct and reporting will be a significant step towards clarifying what is appropriate use of the methods and what is not. This should be followed by the development of a critical appraisal tool to support end users of systematic reviews reporting network meta-analysis.

## Background

Pairwise meta-analysis remains by far the most commonly used method of analysis within systematic reviews of healthcare interventions [1], outnumbering the use of network meta-analysis by more than 20:1, but the use of network meta-analysis (NMA) methods, including mixed treatment comparison (MTC), is increasing rapidly [2]. These methods combine direct and indirect evidence to address the frequent absence of randomised trials that directly compare all the interventions of interest.

International consensus standards for the reporting of systematic reviews and meta-analyses were developed and published as the Quality of reporting of meta-analyses (QUORUM) statement in 1999 [3] and updated in 2009 to become the Preferred Reporting Items for Systematic Reviews and Meta-Analyses (PRISMA) statement [4]. The PRISMA statement mentioned meta-analyses that combine direct and indirect comparisons but prior to 2015 did not contain recommendations for reporting that were specific to NMA methodology. For standards of conduct of systematic reviews, the PRISMA statement directed readers to the guidance published by The Cochrane Collaboration [5] and the Centre for Reviews and Dissemination [6], but each contains very limited guidance on the use of NMA methodology in systematic reviews. A basis for standards of conduct of NMA can be found in the National Institute for Clinical Excellence (NICE) Decision Support Unit’s Evidence Synthesis Technical Support Documents (TSDs) [7] and in reports on the interpretation and conduct of MTCs published in 2011 by the International Society for Pharmacoeconomics and Outcomes Research (ISPOR) [8, 9]. In 2014, Hutton [10] reported on the development of an extension to the PRISMA statement to cover the reporting of NMA and this extension was published in 2015 [11].

In a recent review, I reported the marked increase of systematic reviews that report MTC or NMA since 2009 and considered potential reasons for this increased use, including accessibility and acceptability of the methods. I also outlined future developments that are needed, such as consensus on standards for conduct and reporting [2]. To explore these themes further, I surveyed the authors of the systematic reviews included in my review, to identify their perceptions of use of NMA methods, particularly the standards for conduct and reporting they think should apply. Abdelhamid [12] surveyed authors of Cochrane Reviews about the use of any indirect comparison method in systematic reviews in 2012. Only 14 % of the Cochrane reviews included in that survey included an indirect comparison analysis, and it was not reported whether any of these were MTC or NMA. Additionally, only 23 % of respondents to that survey had actually ever used any indirect comparison method. The survey reported here was not limited to Cochrane review authors and appears to be the first of authors who have published systematic reviews that report specifically MTC or NMA.

This qualitative study was undertaken to identify authors’ perceptions of the use of NMA and of what standards for conduct and reporting should apply.

## Methods

The post-positivist research paradigm was adopted in undertaking this qualitative survey. This is an interpretive approach, which assumes that reality is subjective so is an appropriate option for exploring peoples’ perceptions, as opposed to the positivist approach that assumes there is an objective reality to be discovered [13]. The methods used to identify eligible systematic reviews are described fully in a previous publication [2]. In summary, Medical Literature Analysis and Retrieval System Online (MEDLINE), MEDLINE In-Process, Excerpta Medica Database (EMBASE), Cumulative Index to Nursing and Allied Health Literature (CINAHL), Database of Abstracts of Reviews of Effects (DARE), the Cochrane Database of Systematic Reviews, and SIGLE were searched for reviews published up to June 2012 in which a meta-analysis had been conducted that combined direct and indirect comparisons among more than two interventions. From 2318 records identified through database searching, 390 full-text articles were assessed for eligibility and 201 of these were included in the qualitative synthesis. One hundred fifty seven of these were published in full and 44 as conference abstracts, posters, or presentations.

The target sample for this survey was one author from each of the 201 systematic reviews included in the qualitative synthesis. The survey was conducted by email. For ease of identifying contact details, I contacted the corresponding author but asked to be informed of any other author they thought would be more appropriate to complete the survey. When I received notification of an email address no longer in use and was unable to identify an alternative contact email address for the corresponding author, I contacted the first named author as the next preference, then second named, and so on. When I identified that a contacted author was responsible for more than one review, I asked them to comment on each review when answering the questions that were specific to the review.

The survey contained eight questions (Appendix 1), which were included in the body of the email, following an introductory explanation regarding the background and purpose of the survey. The email informed authors that their responses could be quoted but would not be attributed and identified the specific reviews each author was being contacted about. Since this was a survey of research authors, using their contact details, which were in the public domain, asking about their research, which is also in the public domain, consent to take part was in the form of their choosing to reply. One reminder was sent to each author who failed to respond and to any author who replied indicating that they would respond but had not done so within 4 weeks or, when applicable, by the specific time they had indicated. The survey was conducted between March and July 2014. .In line with National Health Service (NHS) guidance, application for research ethics approval was not required for this survey and I did not apply in advance of the survey for ethics approval from my academic institution but discovered later that I should have done. Following submission of an application, the Research Ethics Manager, Medical Sciences issued a confirmation letter that my study did not raise any concerns regarding the institution’s policies for ethics approval and approval would have been given if my application had been submitted in advance of the survey.

I used an Excel spreadsheet to record, for each review, approaches to and responses from authors. Each review was categorised by date of publication, whether it was published in full or as a conference abstract and whether it was reported within a primarily methodological paper. Authors’ responses were copied into an Excel spreadsheet before being imported to QSR Nvivo 10 for qualitative analysis.

## Results

**Fig. 1:**
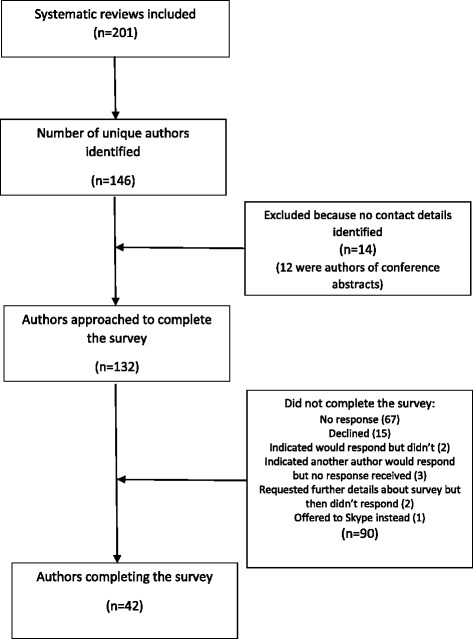
Flow diagram of authors contacted and outcomes

Figure 1 shows the number of unique authors identified and contacted and the outcomes of contact. All ten authors for whom I could not identify a valid email address were authors of one or more conference abstracts. The 42 authors completing the survey is a response rate of 32 %. These authors were responsible for 54 (34 %) of the 157 fully published systematic reviews and 5 (11 %) of the 44 reviews that were only available as conference abstracts. Of the responses, 93 % related to reviews published from 2009 onwards; these constituted 88 % of all included reviews. None of the completed surveys related to any of the four primarily methodological papers.

### Why was the method chosen?

Most responses (28) cited the lack of direct comparison data for all the interventions of interest as the reason for conducting a NMA. Other themes were the desire to rank interventions, the wish to try a new method due to interest generated by other publications, and the view that it improved the strength of the evidence. Comments received included the following:

lack of evidence directly comparing most of the drugs of interest

we wanted to incorporate all available evidence from all trials whether or not they directly compared all treatments simultaneously

Since we were comparing several competing interventions, a methods for ranking the interventions was intuitively preferable to preparing a series of pairwise comparisons

The estimates of indirect comparisons can strengthen and make more accurate the results of direct comparisons when indirect and direct evidence are combined in mixed treatment comparisons.

### Would the author use the same method if repeating the review?

All but one author would use the method again. That author specified the following reason for this,

Because the direct result contradicted the network analysis

Two authors stated they would carry out both direct and indirect meta-analyses,

we indeed plan to perform a network MA again. But we will first perform a standard MA, as this method is more accepted, and then a NMA.

and one qualified the decision,

We would search the literature for direct comparisons, then decide whether an indirect comparison was still necessary

Others explained their reason for using the method again,

it enables us to answer the questions we would otherwise not be able to answer, that is, evaluating the comparative effectiveness of these agents

there is no other approach that would allow simultaneous comparison of several interventions

### Was any reporting guideline followed?

More than half the responding authors (24) stated that they had not followed any reporting guideline. Most [11] of the other 17 respondents mentioned PRISMA [4] but commented that it does not have content specific to this method. Other responses mentioned publications by ISPOR [8, 9], NICE [7], or the Australian Pharmaceutical Benefits Advisory Committee (PBAC) [14]. Some authors stated that they followed the publishing journal’s own guideline or were guided by what other authors had reported when publishing NMA.

### What are essential details to report?

Several authors [10] specifically commented that the same details as recommended for pairwise meta-analysis should be included,

The paper should include all the elements required by the PRISMA guidelines for standard MA

Others emphasised the overall purpose of reporting,

Detailed description of methods used that would allow replication of the MTC

Some think that data files should be provided,

All data in a separate file

Raw data from the trials so that the analysis can be replicated

The detailed reporting elements proposed by authors are summarised in Table 1 (methodological) and Table 2 (results).

**Table 1:** Summary of proposed essential methodological details to be reported

A clear research question
Search details—including terms, databases, period, language restrictions
Inclusion/exclusion criteria including whether study quality was assessed and by what method
Assumptions *The main issue for MTCs to be valid is the consistency assumption. Very often there is not enough information on the included studies to determine whether the consistency assumption is actually reasonable.* *Assessment that the studies are comparable. e.g. consistent endpoint definitions, differences in patient populations or study settings are not expected to influence the treatment effect. Explicitly state and discuss assumptions of the analysis.*
Detailed statistical analysis plan, including software; Bayesian/Frequentist method; random/fixed effects model; code; co-variates; handling of missing data; pooling of data; choice of priors; assessment of heterogeneity; and assessment of consistency *sensitivity analysis of the impact of analyst assumptions is also very important.*

**Table 2:** Summary of proposed essential result details to be reported

Details of included studies *numbers of included studies for each direct comparison; numbers of subjects for each treatment* *study bias assessment* *the quality assessment of included studies*
Details relating to the network *Network diagram including the number of trials included in each link* *Sources of heterogeneity must be assessed and the impact of heterogeneity must be analysed.* *Evaluation of the “confidence” in the network (amount of evidence, homogeneity, consistency)* *How good a fit the chosen model is to the data set.*
Details relating to effect estimates *point estimates and confidence/credible intervals* *95 % credible intervals/probability intervals must be included when reporting the effect estimate* *absolute effect of each intervention [when reporting input parameters of economic modelling]* *reporting of estimates and variances of the direct comparisons that form the indirect comparison* *Comparison of results from direct evidence with results from NMA* *Sensitivity analyses if necessary, and an explanation of the differences compared with standard MA* *The key information needed is to provide separately the direct and indirect estimates, and try to provide the quality of evidence supporting each rankings [perhaps rankograms as well] and probabilities of each intervention being best.* *For Bayesian mixed treatment comparisons: Probability Rankograms and Surface Under Cumulative Ranking Curve* *Well reasoned sensitivity analysis, including/excluding different data sources*.

### Does any reporting guideline contain the essential details?

The most frequent response [11] to this question was that there was not such a guideline. A few authors mentioned the planned extension to the PRISMA statement. Of those who named a guideline, the most frequently named, in decreasing order, were ISPOR [8, 9], the NICE TSD series [7], and PRISMA [4].

### What developments are needed to support conduct, reporting, and use of the methods?

Most authors (30) stated that development and promotion of such guidelines is needed,

development of guidelines that include all details necessary to the performance of an indirect comparison

Specific guidelines on how to properly report and interpret the results of a network meta-analysis should also be developed

Each of the following themes was raised by some authors:

#### Methodological

more evidence that ranking probability is an accurate measure

I believe that MTCs should only be presented as comparative effect estimates but never as a ranking of interventions.

Risk of bias tools

incorporate methods such as I ^2^ for NMA and the incorporation of inconsistency as in the design-by-treatment model.

a way to present results in a simple way (perhaps not in print, but rather using animation) to include absolute and relative effects, risk of bias, for each outcome, with info about precision, heterogeneity and coherence, and overall quality of evidence.

objective way of testing for convergence

inclusion of evidence from observational studies is probably an area for development to make use of all available evidence

#### Software

more easy-to-use software would be good

not requiring a higher degree in statistics to make use of it

Improvement of available software – at the moment WinBUGS and R are considered by many reviewers too complex to use in routine systematic reviewing. New/improved software needs to be more user-friendly and less time consuming.

#### Training

currently few persons are able to carry out the statistical computations, and this is limiting the diffusion of network meta-analyses

The statistical methodology should be diffused much more than it is today

#### Presentation of results

What is needed the most is a way to communicate the findings to readers/users.

#### Support

access to specialist statisticians

Several authors [11] expressed concerns about widening use of the methods and evidence produced by it:

I am concerned that accessibility may be at the expense of thinking

my concern is the unthinking use of any method without a statistical appreciation of the model(s) and the assumptions

Particularly when they use rankings, the information is often misleading because readers tend to focus on the top intervention

Nowadays people “believe” in MA as if it were the absolute truth, not understanding that there are good quality and poor quality MA. And same with network MA, but the risk is even higher in this latter case.

These methods are becoming increasingly popular, and there are many examples of poorly performed analyses

There are many subtleties and underlying assumptions in performing such an analysis, and increasingly there are many “automated” analysis, where data are pulled from papers and fed into computer programs, with poor assessment or even identification of assumptions.

those publishing indirect comparisons understand how to assess their quality

stricter editorial processes to ensure adherence to systematic review, statistical and reporting standards

those utilizing indirect comparisons in their decision-making are able to recognize when they are performed correctly and can be confident in their results

The problem of network meta-analyses and indirect comparisons is that the statistical methods are difficult for most readers to understand or reproduce. Therefore, it is a black box.

I believe the use of it should be determined via the careful judgement of whether it is really needed, not just 'encouraged' regardless of the research question and the level of parameter heterogeneity.

### What would encourage more use of the methods by systematic reviewers?

Guidelines were again a common theme in response to this question, alongside greater perceived acceptability of use of these methods, particularly through endorsement by key organisations and increased likelihood of publication,

Guidelines would help

promotion of guidelines etc to Cochrane review groups

Uptake would also be increased if HTA organisations other than NICE gave explicit statements on the acceptability of indirect comparisons and NMA and issued guidance.

Less scepticism by the methods community.

Increased likelihood of publication

Stronger journal policies encouraging its use.

Other themes were training, software development, and access to statistical expertise:

#### Training

Reviewers need to be educated in the proper performance and reporting of indirect comparisons. More broadly, they need to be educated that such methods exist and can be used to derive answers unattainable by other methods.

The knowledge of the method, of its possibilities, aims and limits would encourage the reviewers to use MTC

Understanding of assumptions and pitfalls to give more confidence in the use of the method.

Better understanding of indirect comparison methods, and their comparability with ‘classical’ meta-analysis. The reviewers need to understand the advantages of such methods, and the fact that most limitations of the indirect comparison methods are indeed limitations of the classical direct comparison methods as well.

#### Software

Software has to become more user-friendly.

easy frequentist software

Standard code for different situations

#### Statistical expertise

Currently, the statistical expertise necessary is the main limitation

### What would encourage more use of the methods by decision makers?

Training was again a clear theme,

Again, education is key. Indirect comparisons can be performed well or poorly, and exposure to well-performed indirect comparisons and education in how to identify one that has been performed poorly will enable decision makers to broaden the array of evidence that they can use to support their decisions.

including tutorial articles explaining the basic premise in non-technical language and individual applications explaining their methods and results in a way that is accessible to wider readers.

There was mixed opinion regarding the desirability of this aspect of increased use.

I would not encourage it for the sake of it. I think there are situations where it would be better avoided, e.g. where there is robust direct evidence.

the methods are complex and it would be very difficult for someone without extensive technical expertise to identify what the flaws are in any given indirect comparison or network meta-analysis, let alone whether the code used in the analysis was correct.

Currently it is seen as a “strange, peculiar” method, and somewhat mistrusted

I think it’s moving in the right direction really – NICE support them, because they’re a ‘necessary evil’ – there’s a well known disconnect between the trial design required for licensing, and that for the evidence base needed for reimbursement and clinical decision making. People maybe still see it as ‘voodoo’ but in general I think we at least in the UK have a reasonably good and established process for selecting the best evidence available to inform decision making.

We currently stand at a crossroads where policy makers are content for indirect comparisons to be undertaken for the purposes of health economic evaluation. However, a fundamental shift in understanding needs to take place so that decision makers (policy makers/clinicians and in time the general public) accept the validity of the estimates of safety/efficacy that come from the analysis in their own right. In particular, indirect comparisons appear to have the same “face validity” to clinicians as pairwise meta-analysis did 15+ years ago.

## Discussion

To my knowledge, this is the first published survey reporting the attitudes of systematic review authors who have used NMA methods to reporting standards for such research.

This survey found that systematic review authors who have used NMA methods did so mainly because of the lack of direct trial data or because of the ability to compare and rank multiple interventions. Authors who have used these methods are mostly inclined to use them again. Their responses demonstrated strong support for adoption of standards for conduct and reporting of NMA. The elements of reporting standards proposed by these authors are substantially consistent with those reported by Hutton [10, 11] as part of the development work for and final publication of the extension of the PRISMA statement. This should augur well for adoption of that extension, suggesting that it will carry the support of authors publishing this form of meta-analysis. Support in principle for the content of a reporting guideline does not however necessarily translate into compliance [15]. Whilst several responders to the survey made the observation that reporting standards should include those required for pairwise meta-analysis, most reported that they had not followed any reporting guideline.

A notable theme arising from the responses was the tension between, on the one hand, a view that the use of NMA should be more easily accessible, particularly in the form of software tools, and on the other, concerns that there is some inappropriate use of the methods which wider use and greater accessibility could exacerbate. This tension prompts the question: what is the ability of the end users (clinicians, commissioners, policy makers) to critically appraise and interpret the results produced using this methodology? Adoption of standards for conduct and reporting will be significant steps towards clarifying what is appropriate use and what is not. I suggest this should be followed by the development of a critical appraisal tool to further support end users.

### Limitations of the survey

The respondents formed a minority of the authors approached and the views of non-respondents are unknown. Nevertheless, there was no apparent bias in those responding in terms of year of publication or in the number or subject of reviews published. Conducting the survey by email afforded limited opportunity to clarify responses and did not allow me to pursue lines of enquiry, so the responses are what authors chose to volunteer and therefore cannot be assumed to represent all their opinions. The author is a part-time research student, researching reporting standards and critical appraisal criteria for network meta-analyses reported in systematic reviews. Survey questions made specific mention of reporting guidelines, which may have influenced the mention of these in responses. My research interests influenced the questions and are likely to have influenced both the selection of responses I have quoted and my overall interpretation of all responses; however, I approached this survey without pre-conceived expectations of what the responses would be but with the hope that the responses might identify specific issues or themes that would be suitable for further exploration.

## Conclusions

Authors demonstrated strong support for adoption of standards for conduct and reporting of network meta-analysis. The elements of reporting standards proposed are consistent with those included in the 2015 PRISMA extension statement. As more widespread use of the methods continues to develop, it should be accompanied by assurance that use is appropriate. Adoption of standards for conduct and reporting will be a significant step towards clarifying what is appropriate use of the methods and what is not. This should be followed by the development of a critical appraisal tool to support end users of systematic reviews reporting network meta-analysis.

## Appendix 1

Survey questions

1. Why did you choose an indirect comparison method for meta-analysis in your review?
2. If you were repeating the review in future, would you choose an indirect comparison method again? If you would not, please explain why.
3. Please name any reporting guideline(s) you followed when reporting the indirect comparison and the review.
4. What, in your opinion, are the essential details to include for accurate and reliable reporting of indirect comparison in a review?
5. Please name any reporting guideline(s) that, in your opinion, includes the essential details for accurate and reliable reporting of indirect comparison in a review.
6. What is (are), in your opinion, the most important development(s) needed to support the conduct, reporting and use of indirect comparison methods?
7. What would encourage further use of indirect comparison methods by reviewers?
8. What would encourage further use of evidence from reviews reporting indirect comparison methods by decision makers (including policy makers, clinical practitioners and the public)?

## Abbreviations

CINAHL: Cumulative Index to Nursing and Allied Health Literature
DARE: Database of Abstracts of Reviews of Effects
EMBASE: Excerpta Medica Database
HTA: Health Technology Assessment
ISPOR: International Society for Pharmacoeconomics and Outcomes Research
MA: meta-analysis
MEDLINE: Medical Literature Analysis and Retrieval System Online
MTC: mixed treatment comparison
NICE: National Institute for Clinical Excellence
NMA: network meta-analysis
PBAC: Pharmaceutical Benefits Advisory Committee
PRISMA: Preferred Reporting Items for Systematic Reviews and Meta-Analyses
QUORUM: Quality of reporting of meta-analyses
SIGLE: System for Information on Grey Literature
TSD: Technical Support Document
